# Mortality Risks among Various Primary Renal Diseases in Children and Adolescents on Chronic Dialysis

**DOI:** 10.3390/jcm7110414

**Published:** 2018-11-05

**Authors:** Hsin-Hsu Chou, Yuan-Yow Chiou, Yee-Hsuan Chiou, You-Lin Tain, Hsin-Hui Wang, Mei-Ching Yu, Chih-Cheng Hsu, Ching-Yuang Lin

**Affiliations:** 1 Department of Pediatrics, Ditmanson Medical Foundation Chia-Yi Christian Hospital, Chiayi 600, Taiwan; edberg.chou@msa.hinet.net; 2 Department of Applied Life Science and Health, College of Human Ecology, Chia Nan University of Pharmacy and Science, Tainan 717, Taiwan; 3 Departments of Pediatrics, Institute of Clinical Medicine, National Cheng Kung University Medical College and Hospital, Tainan 704, Taiwan; yuanyow@mail.ncku.edu.tw; 4 Department of Pediatrics, Kaohsiung Veterans General Hospital, Kaohsiung 813, Taiwan; chysn@ms6.hinet.net; 5 Department of Medical Technology, Fooyin University, Kaohsiung 831, Taiwan; 6 Division of Pediatric Nephrology, Chang Gung Memorial Hospital-Kaohsiung Medical Center, Kaohsiung 833, Taiwan; tainyl@hotmail.com; 7 Department of Pediatrics, Taipei Veterans General hospital, Taipei 112, Taiwan; hhwang@vghtpe.gov.tw; 8 Division of Pediatric Nephrology, Chang Gung Memorial Hospital-Linko Medical Center and Chang Gung University, Taoyuan 333, Taiwan; yumeiching@gmail.com; 9 Institute of Population Heath Sciences, National Health Research Institutes, Miaoli 350, Taiwan; cch@nhri.org.tw; 10 Institute of Clinical Medicine, National Yang-Ming University, Taipei 112, Taiwan; 11 Department of Health Services Administration, China Medical University, Taichung 404, Taiwan; 12 Clinical Immunological Center, China Medical University Medical College and Hospital, Taichung 404, Taiwan

**Keywords:** mortality risk, primary renal disease, end-stage renal disease, chronic dialysis, children and adolescents

## Abstract

There is little information available on the association between primary renal disease (PRD) and long-term mortality in the pediatric dialysis population. The objective of this study was to explore mortality risks in children and adolescents on chronic dialysis, specifically focused on the risk of various PRDs. The study cohort included children and adolescents with end-stage renal disease (ESRD) (aged < 20 years) who had received dialysis for at least 90 days between 2000 and 2014 and were identified from Taiwan’s National Health Insurance medical claims. A total of 530 children and adolescents were included in the study. The median age of the included patients was 13.6 years and 305 (57.5%) patients were males. One hundred and seven patients died during the follow-up period and the median survival time was 6.0 years. Mortality was highest in the youngest patients. For patients with the following PRDs, mortality was significantly higher than that in patients with primary glomerulonephritis: secondary glomerulonephritis (adjusted hazard ratio (aHR): 2.50; 95% confidence interval (CI): 1.03–6.08), urologic disorder (aHR: 4.77; 95% CI: 1.69–13.46), and metabolic diseases (aHR: 5.57; 95% CI: 1.84–16.85). Several kinds of PRDs appear to have high mortality risks in the pediatric dialysis population. These differences in mortality risk highlight the importance of the focused clinical management of these high-risk subgroups.

## 1. Introduction

End-stage renal disease (ESRD) in children is a chronic condition that is associated with high mortality and specific problems, such as impaired growth and psychosocial impact, which lead to impaired quality of life [1,2]. Renal replacement therapy (RRT) is usually required to maintain life or to bridge the time before kidney transplantation. Although survival in these children has increased substantially over the past decades, mortality remains very high, approximately 55–150 times higher than in the general pediatric population [3,4].

Several risk factors for survival have been identified in ESRD children on chronic dialysis, including age at initial dialysis, initial dialysis modality, era of dialysis, duration of dialysis, transplantation, and presence of comorbidities [5,6,7,8]. Although it is generally believed that survival varies by primary renal disease (PRD), few studies have reported the association between long-term survival in the pediatric dialysis population and PRD. The United States Renal Data System (USRDS) showed that children with glomerulonephritis and hereditary or congenital disease had longer 5-year survival than those with secondary glomerulonephritis or vasculitis [3]. Patients with congenital anomalies of the kidney and urinary tract (CAKUT) have a superior survival among all ESRD patients [6]. However, there are great varieties within the etiologies of PRD. Mortality may vary between patients with renal hypodysplasia, reflux nephropathy, and urologic disorders in those with CAKUT. Identification of subgroups of patients at risk is essential for those caring for pediatric patients on dialysis and might improve the long-term survival and quality of life for both these children and their parents.

This study therefore aims to (i) describe the mortality in Taiwanese children and adolescents on chronic dialysis; (ii) explore the mortality risk stratified by age, sex, PRD, calendar year at dialysis initiation, initial dialysis modality, and presence of comorbidities; and (iii) compare the differential mortality risk in prespecified subgroups of PRD in pediatric dialysis patients.

## 2. Experimental Section

### 2.1. Data Source

This study was a population based, nationwide cohort study using medical claim data from Taiwan’s National Health Insurance (NHI) medical claims and National Death Registry between 1 January 2000, and 31 December 2014. We established the longitudinal medical history of each patient by linking both claims datasets through the civil identification number unique to each beneficiary and their date of birth. Up to 99% of the Taiwan’s 23 million residents receive medical care through the Taiwan’s NHI program [9]. The NHI system defines several major illnesses, such as ESRD, as “catastrophic illnesses” and provides guidelines and regulations for the insured person to apply for a catastrophic illness certificate. The Bureau of NHI performs routine validations of the diagnoses by reviewing the original medical charts of all the patients who apply for catastrophic illness certificates. In this study, all ESRD patients were identified from the catastrophic illness registry data, which is a subset of the NHI database. We used both inpatient and outpatient medical claims data from the NHI program, which provided information on diagnoses and procedures for each clinical visit and admission. Information on death was obtained from the National Death Registry. In Taiwan, it is a legal requirement that all deaths be registered within 15 days. Information from the datasets and links between the datasets have been validated, and a high level of completeness and validity has been observed for most of its components [10,11]. Data released to the public for research purposes are deidentified and encrypted, so this study was exempted from full review by the Institutional Review Board of Chia-Yi Christian Hospital (CYCH-IRB No.: 2018050).

### 2.2. Study Population

Patients who were younger than 20 years of age at the initiation of dialysis, either hemodialysis (HD) or peritoneal dialysis (PD), and who had received dialysis for more than 90 days were included. The initial treatment modality was defined as treatment at day 90 because some patients were started on HD initially to prepare for PD. Patient information was extracted including date of birth, sex, date of initiation of dialysis, dialysis modality, PRD, number of morbidities, and events such as death or changes in treatment modalities. Age at dialysis initiation was divided into 4 groups: 0–1, 2–5, 6–12, and 13–19 years of age. The calendar year of dialysis initiation was categorized as 2000–2004, 2005–2009, and 2010–2014. Death was assigned to a patient’s initial dialysis modality regardless of dialysis modality changes. Patients were censored when reaching the end of the observation period, or when received renal transplantation, whichever came first.

PRDs were identified from both inpatient and outpatient claims, using codes from the clinical modification of the International Classification of Diseases, 9th edition (ICD-9-CM, see Supplemental Table S1 in the supplementary material). We included only those patients for whom diagnosis were recorded during at least one hospital admission or during more than 2 outpatient clinic visits before the initiation of dialysis. PRDs were classified as glomerular and nonglomerular diseases [12] and were further divided into several categories with modification from the European Renal Association-European Dialysis and Transplant Association (ERA-EDTA) grouping of PRD classification codes for children [13] and USRDS classification for primary cause of ESRD in children [3]. PRDs with a glomerular cause were divided into 5 categories: primary glomerulonephritis, nephrotic syndrome, secondary glomerulonephritis, chronic glomerulonephritis (CGN), and other glomerular diseases. We distinguished chronic glomerulonephritis group from primary or secondary glomerulonephritis for analysis since patients in this group are usually characterized by an insidious onset, and asymptomatic hematuria or proteinuria could be the only presenting sign. Some of these patients may not undergo a renal biopsy due to advanced stage CKD and overt renal atrophy. In contrast, patients with primary or secondary GN more often display typical clinical symptoms/signs of GN, and most receive a definitive diagnosis by renal biopsy. PRDs with a nonglomerular cause were divided into 8 categories: renal hypodysplasia, cystic kidney disease, reflux nephropathy, obstructive uropathy, urologic disorders, ischemic, metabolic, and other nonglomerular diseases. The identification of PRD was reviewed independently by two pediatric nephrologists. Patients with none or more than one PRD diagnosis subscribing to one of the groups listed above were discussed among all the investigators, to be categorized as best as possible. Comorbidities that were diagnosed during dialysis, including diabetes, hypertension, ischemic heart disease, congestive heart failure, chronic liver diseases and cirrhosis, stroke, and malignancy were identified by ICD-9-CM codes (Supplemental Table S2).

### 2.3. Statistical Analysis

The primary outcome of study was all-cause death while on dialysis. Baseline characteristics, dialysis modalities and survival time of patients with either glomerular or nonglomerular diseases were compared. Continuous variables that were nonnormally distributed are presented as the median and interquartile range (IQR, the range between the 25th and 75th percentile) and were compared between the glomerular and nonglomerular groups by the Mann–Whitney U test. Categorical variables are presented as counts and percentages and were compared by Pearson’s *χ*^2^ test or Fisher’s exact test, as necessary. After stratification by age, sex, primary cause of ESRD, dialysis modality, and number of comorbidities, all-cause mortality rates and hazard ratios (HRs) were calculated. Survival curves were displayed to describe the crude and adjusted cumulative mortality rates among different PRDs and compared by Kaplan–Meier estimates and log rank test. Mortality risk differences among different PRDs were estimated using multivariate Cox proportional hazards model. All analyses were adjusted for age group, sex, initial dialysis modality, calendar year of dialysis initiation, and number of comorbidities. The crude and adjusted hazards ratios (HRs) and the corresponding 95% confidence intervals (CIs) were calculated. We adjusted for age groups (ages 0–1, 2–5, 6–12, and 13–19 years) instead of using age as a continuous variable because age did not show a linear relationship with mortality. All statistical analyses were performed using SAS software version 9.2 (SAS Institute Inc., Cary, NC, USA). A two-tailed *p* < 0.05 indicated statistical significance.

## 3. Results

### 3.1. Patients Characteristics

Between 1 January 2004, and 31 December 2014, we identified 530 children and adolescents younger than 20 years of age who were starting RRT on dialysis for more than 90 days. Patient characteristics by different PRDs are provided in Table 1. Of these patients, 57.6% were male and the median age at initiation of dialysis was 15.6 years (IQR 12.2–17.2 years). Among the primary causes of ESRD in children and adolescents, 250 patients (47.2%) had glomerular diseases, 230 patients (43.4%) had nonglomerular diseases, and 50 patients (9.4%) had an unknown etiology. Most of the patients (61.1%) received PD as the initial dialysis modality and the proportion of PD did not differ between those with glomerular and nonglomerular diseases (59.2% versus 64.4%). Approximately half of the patients received kidney transplants (275 patients, 51.9%) before the end of the observation period, and the proportion of kidney transplants was higher among patients with glomerular diseases than among those with nonglomerular diseases (68.0% in glomerular diseases versus 37.4% in nonglomerular diseases).

### 3.2. Overall Mortality in Patients on Dialysis

A total of 107 deaths occurred during 3397.9 patient-years (py), which is equivalent to a crude mortality rate of 31.5 deaths per 1000 py during the follow-up period, while censoring for transplantation (Table 1). The crude mortality rate was higher among patients with nonglomerular diseases than among those with glomerular diseases (mortality rate 43.7 deaths per 1000 py in nonglomerular diseases versus 23.4 deaths per 1000 py in glomerular diseases). The median survival time was 6.0 years and the survival time was also longer among patients with nonglomerular diseases than among those with glomerular diseases (survival time 7.0 years in glomerular diseases versus 4.5 years in nonglomerular diseases).

### 3.3. Mortality Risk in Patients on Dialysis

The mortality rates and associated HRs after stratification by age groups, sex, primary cause of ESRD, calendar year of dialysis initiation, initial dialysis modality, and number of comorbidities are demonstrated in Table 2. Mortality was highest in the youngest patients (age of 0–1 year, 266.7 deaths per 1000 py) and decreased as the age at dialysis initiation increased. After adjustment for the abovementioned potential confounders, the patients aged 0–1 year still had a remarkably high risk of death compared with patients aged 13–19 years (age of 0–1 year versus age of 13–19 years: adjusted hazard ratio (aHR) 11.45, 95% CI 4.74–27.67). Both patients aged 2–5 years and those aged 6–11 years also showed significantly increased risks of death compared with patients aged 13–19 years. The overall adjusted mortalities did not differ among patients with different sex, glomerular or nonglomerular diseases, calendar year at dialysis initiation, initial dialysis modality, and number of morbidities.

### 3.4. Mortality Rates and Differential Mortality Risks among Patients with Primary Renal Diseases

**Figure 1:**
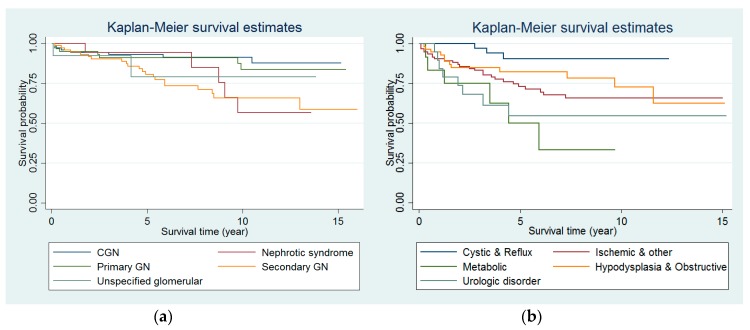
Kaplan–Meier graph of survival rates among pediatric dialysis patients in Taiwan according to various primary renal diseases: (**a**) glomerular disease; (**b**) nonglomerular disease. (CGN, chronic glomerulonephritis; GN, glomerulonephritis).

Among the glomerular disease group, the most common disease was CGN, which occurred in 74 patients (29.7% of glomerular disease patients), followed by secondary GN (28.9%), primary GN (24.5%), and other glomerular etiologies (Table 3). Among the nonglomerular disease group, the most common disease was renal hypodysplasia, which occurred in 46 patients (20.0% of nonglomerular disease patients), followed by reflux nephropathy, ischemic disease, urologic disorders, cystic kidney diseases, obstructive uropathy, and metabolic disorder. The long-term survival among patients with different PRDs is shown in Figure 1. The survival rates were significantly different among patients with various PRDs both in the glomerular and nonglomerular disease groups (log rank test: *p* = 0.006 in the glomerular disease group, and *p* = 0.031 in the nonglomerular disease group). We studied the association between different PRDs among patients on chronic dialysis and overall mortality. After adjustment for age, sex, PRDs, calendar year of dialysis initiation, initial dialysis modality, and number of comorbidities, patients with secondary GN, urologic disorders, and metabolic diseases had significantly greater mortality risk than patients with primary GN (secondary GN versus primary GN: aHR 2.50, 95% CI 1.03–6.08; urologic disorders versus primary GN: aHR 4.77, 95% CI 1.69–13.46; metabolic diseases versus primary GN: aHR 5.57, 95% CI 1.84–16.85; see Table 4).

## 4. Discussion

To our knowledge, this population-based study is the largest cohort study to report the all-cause mortality in East Asian, specifically Taiwanese, pediatric ESRD patients on chronic dialysis. It also examined the mortality risks, specifically focused on PRDs, in association with long-term survival in these patients. To our knowledge, this is the first clinical study to comprehensively evaluate the roles of different PRDs in association with survival in pediatric ESRD patients on chronic dialysis. Patients with secondary GN, urologic disorders, and metabolic diseases had greater mortality risk than patients with primary GN while on chronic dialysis. In addition, we also identified children who started dialysis at younger age, in particular, children younger than 1 year old had a much higher mortality rate than young adolescents on chronic dialysis. These differences in mortality risks highlight the importance of focused clinical management in these high-risk subgroups.

The overall mortality rate in Taiwanese children on chronic dialysis was generally comparable with the mortality rates reported in other nationwide reports, which has ranged from 17 to 44 deaths per 1000 py during the same approximate period of time. USRDS data reported a mortality rate of 44 deaths per 1000 py in patients younger than 21 years old who started dialysis between 2000 and 2010. In the most recent report, the ERA-EDTA reported an overall mortality rate of 28.0 deaths per 1000 py in patients on dialysis during the first 5 years of dialysis treatment between 2000 and 2013, while censoring for transplantation [5]. The mortality rate in pediatric Japanese patients with ESRD younger than 20 years old was 18.2 per 1000 py during the period between 2006 and 2011 [14]. The reason for the differences in mortality rates between countries is not clear. Part of this difference might be explained by variations in the ages of patients starting RRT, racial predisposition, differences in PRDs and the choices of RRT modalities, socioeconomic status or other unknown reasons [5,6,8,15]. Besides, difference in the incidence of primary renal disease may play a role in the differences in mortality rates between countries. Glomerulonephritis and CAKUT form the most common etiologies of renal disease in children, however, in our previous study [16], glomerular diseases account for nearly half of patients with chronic kidney disease in Taiwan, where glomerulonephritis is present in only 20–30% of patients in Europe [5] and 30–40% in the United States [3].

Registry data consistently shows a significant higher mortality rate in infants starting dialysis than in older children. Mortality risk is approximately four times higher in children younger than 5 years of age at dialysis initiation than in older children, and 1.5 times higher in children aged 5–12 years old at dialysis initiation than in older children [4,6,7]. Our study also revealed that children in the youngest age group at initiation of dialysis (less than 1 year old) had the worst survival rate, with a more than 11-fold increased risk of death compared with those in the adolescent group. Technical challenges due to small body size and difficult vascular access, high risk of infection, difficulties in providing adequate nutrition which results in growth retardation, and a high prevalence of comorbidities may partially explain the high mortality in newborn and infants receiving dialysis treatment.

Our study showed that the all-cause mortality in patients with secondary GN on chronic dialysis was higher than in patients with primary GN. The most prevalent PRD of patients in the secondary GN group was lupus nephritis (77%) and the main cause of death was cardiovascular disease (57%), followed by infection (29%). These findings were comparable with those of the USRDS data which reported a five-year survival rate of 78% and a 2-fold increased risk of death compared with other pediatric patients with ESRD; the most common causes of death were cardiovascular disease and cardiac arrest [17]. An increased inflammatory state that leads to atherosclerosis may account for the much younger age at death in patients with ESRD secondary to lupus nephritis than in other patients [18]. Extrarenal manifestations of systemic lupus erythematosus (SLE) including cardiac involvement and hemolytic anemia, dyslipidemia, and hypertension, and the use of medications to treat lupus nephritis, such as glucocorticoids, may also contribute to increased cardiovascular death [19,20]. Clinician should be aware that the diagnosis of SLE alone may be associated with an increased risk of death in patients with ESRD.

The mortality rates were significantly higher in patients with urologic disorders and metabolic diseases than in patients with primary GN. Most patients with urologic disorders in our cohort had neurogenic bladder (75%) and the main cause of death was infection (67%). Indeed, there is a clear association between bladder dysfunction and urinary tract infection (UTI) [21]. A previous study revealed that hospitalization related to UTIs was positively associated with risk of death on dialysis in patients with ESRD and spinal bifida [22]. Proper urological and urinary bladder management is imperative in patients with neurogenic bladder, including concomitant vesicoureteral reflux, bowel and bladder dysfunctions and potential behavioral and neurodevelopmental issues. Prevention and treatment of symptomatic UTI requires a multimodal approach that focuses on bladder management as well as accurate diagnosis and appropriate antibiotic treatment.

There were substantial diversities in the primary diseases affecting chronic dialysis patients with metabolic disorders in this study, including renal calculi, nephrocalcinosis, Fabry disease and other enzymopathies or genetic disorders. Cardiovascular disease was the most common cause of death in these patients. Data on the outcomes and survival of these children remain limited, however, several metabolic diseases, such as Fabry disease and primary oxalosis, have been reported to have poor outcomes [23,24]. Failure to thrive, developmental delay and multiple organ involvement especially neurologic disease and cardiomyopathy are not uncommon, which may partly explain the high risk of mortality in these patients.

This study was based on a large sample from universal medical insurance database in Taiwan with a long follow-up period. This allowed identification of a study sample free from selection bias and of sufficient size to document outcomes. However, several limitations should be noted. First, the identification of PRD was mainly based on claims data and ICD-9-CM diagnosis codes, which could potentially lead to disease misclassification. However, such misclassification should be minimal, given that the identification of PRD in each patient was reviewed independently by two pediatric nephrologists and disagreement was resolved by consensus among all investigators. Moreover, regular chart review and cross-checking mechanisms are conducted by Taiwan’s NHI Bureau to facilitate the accuracy of coding [25]. Second, detection of incident cases of ESRD was not complete because the data from patients who died before receiving RRT for less than 90 days was not included. This may have decreased the HR as the sickest patients were not included in the analysis. Third, there was potential for residual confounding because laboratory data, specific data on dialysis adequacy, medical prescriptions, and socioeconomic status were not available in this study. These unmeasured risk factors might have biased the results if they were differentially distributed in patients versus comparisons.

## 5. Conclusions

Mortality risk varies between different PRDs in children and adolescents on chronic dialysis. We demonstrated an increased mortality risk in patients with secondary GN, urologic disorders and metabolic diseases compared with that in patients with primary GN. The identification of these vulnerable subgroups in the pediatric dialysis population allows early recognition and intervention for these risk factors for mortality, especially cardiovascular disease and infection. Furthermore, a multidisciplinary approach for identifying potential comorbidities in complicated cases and early preparation for kidney transplantation may improve survival in children on chronic dialysis.

## Figures and Tables

**Table 1:** Characteristics of children with end-stage renal disease (ESRD), stratified by glomerular and nonglomerular diseases, 2000–2014.

	Overall (*n* = 530)	Glomerular (*n* = 250)	Nonglomerular (*n* = 230)	Unknown (*n* = 50)	*p*-Value
Age at dialysis initiation, median (IQR), years	15.6 (12.2, 17.2)	16.5 (13.0, 17.0)	14.5 (9.1, 14.9)	15.8 (13.6, 17.0)	<0.001 **
Male sex, No. (%)	305 (57.6%)	136 (54.4%)	144 (62.6%)	25 (50.0%)	0.101
Initial dialysis modality, No. (%)					0.378
Hemodialysis	206 (38.9%)	102 (40.8%)	82 (35.7%)	22 (44.0%)	
Peritoneal dialysis	324 (61.1%)	148 (59.2%)	148 (64.4%)	28 (56.0%)	
Survival status, Yes, No. (%)	423 (79.8%)	208 (83.2%)	175 (76.1)	40 (80.0%)	0.152
Survival time, median (IQR), years	6.0 (3.6, 8.5)	7.0 (4.8, 9.6)	4.5 (2.3, 7.0)	7.5 (5.0, 10.2)	<0.001 **
Crude mortality rate at dialysis initiation, 2000–2014	31.5 per 1000 py	23.4 per 1000 py	43.7 per 1000 py	28.9 per 1000 py	0.024 **
Dialysis modality at the end of observation					<0.001 **
Hemodialysis	179 (33.8%)	63 (25.2%)	93 (40.4%)	23 (46.0%)	
Peritoneal dialysis	76 (14.3%)	17 (6.8%)	51 (22.2%)	8 (16.0%)	
Kidney transplant	275 (51.9%)	170 (68.0%)	86 (37.4%)	19 (38.0%)	
Number of comorbidities, No. (%)					0.050
None	256 (48.3%)	116 (46.4%)	109 (47.4%)	31 (62.0%)	
1	229 (43.2%)	119 (47.6%)	95 (41.3%)	15 (30.0%)	
≥2	45 (8.5%)	15 (6.0%)	26 (11.3%)	4 (8.0%)	

* Continuous data are presented as the median (interquartile range). Categorical variables are expressed as counts (percentages).** Indicates a significan difference between three groups; IQR: interquartile range; py: patient-year; No., number.

**Table 2:** Adjusted all-cause mortality rates and hazard ratios (HRs) for mortality, stratified by patient age, sex, dialysis modalities, and number of comorbidities among end-stage renal disease (ESRD) patients, 2000–2014.

	No. of Patients	Patient-Years (py)	No. of Deaths	Deaths per 1000 py	Unadjusted HR (95% CI)	Adjusted HR * (95% CI)
Age at dialysis initiation						
0–1	9	26.25	7	266.67	11.53 (5.16–25.76)	11.45 (4.74–27.67)
2–5	19	67.08	8	119.26	5.12 (2.40–10.93)	5.43 (2.45–12.04)
6–12	116	682.92	37	54.18	2.63 (1.70–4.07)	2.57 (1.62–4.07)
13–19	336	2283.58	45	19.71	Reference	Reference
Sex						
Male	280	1808.67	52	28.75	0.81 (0.54–1.21)	0.95 (0.62–1.44)
Female	200	1251.17	45	35.97	Reference	Reference
Primary cause of ESRD						
Glomerular	250	1701.92	42	24.68	Reference	Reference
Nonglomerular	230	1257.92	55	43.72	1.74 (1.17–2.61)	1.26 (0.82–1.94)
Calendar year at dialysis initiation, per 1000 py						
2000–2004	177	1580.0	43	27.22	Reference	Reference
2005–2009	170	1021.67	36	35.24	1.12 (0.71–1.76)	1.01 (0.63–1.60)
2010–2014	133	458.17	18	39.29	1.08 (0.61–1.92)	1.04 (0.58–1.86)
Initial dialysis modality, No. (%)						
Hemodialysis	184	1248.83	37	29.63	0.95 (0.63–1.43)	1.38 (0.88–2.14)
Peritoneal dialysis	296	1811.0	60	33.13	Reference	Reference
No. of comorbidities						
0	225	1567.17	44	28.08	Reference	Reference
1	214	1280.08	41	32.03	1.09 (0.71–1.67)	1.09 (0.70–1.67)
≥2	41	212.58	12	56.45	1.88 (0.99–3.56)	1.57 (0.81–3.03)

CI, confidence interval; ESRD, end-stage renal disease; HR, hazard ratio; py, person-year. No., number. * Estimated from a multivariate Cox proportional hazards model with adjustment for age group, sex, primary cause of ESRD, initial dialysis modality, calendar year of dialysis initiation, and number of comorbidities.

**Table 3:** Number and proportion of patients according to different primary renal diseases (PRD).

Primary Renal Disease	N (%)
**Glomerular**	249 (47.0%)
Primary glomerulonephritis	61 (11.5%)
Nephrotic syndrome	24 (4.5%)
Chronic glomerulonephritis	74 (14.0%)
Secondary glomerulonephritis	72 (13.6%)
Lupus nephritis	59 (11.1%)
HSP	5 (0.9%)
HUS	6 (1.1%)
Other secondary glomerulonephritis	2 (0.4%)
Others	18 (3.4%)
**Nonglomerular**	230 (43.4%)
Renal hypodysplasia	46 (8.7%)
Renal agenesis/dysplasia	43 (8.1%)
Renal atrophy of unknown cause	3 (0.6%)
Reflux nephropathy	32 (6.0%)
Obstructive uropathy	12 (2.3%)
Urologic disorder	20 (3.8%)
Neurogenic bladder	15 (2.8%)
Other urologic disorder	5 (0.9%)
Cystic kidney disease	13 (2.5%)
ARPKD	4 (0.8%)
ADPKD	1 (0.2%)
Other cystic kidney disease	8 (1.5%)
Ischemic	30 (5.7%)
Asphyxia/hypoxia	15 2.8%)
Shock or severe sepsis	10 (1.9%)
Other ischemic disorder	5 (0.9%)
Metabolic	12 (2.3%)
Urolithiasis or nephrocalcinosis	5 (0.9%)
Disorders of amino acid transport	3 (0.6%)
Fabry disease	2 (0.4%)
Other enzymopathy	2 (0.4%)
Others	65 (12.3%)
Multiple anomalies	18 (3.4%)
Wilms’ tumor	5 (0.9%)
Hypertensive chronic kidney disease	19 (3.6%)
Other nonglomerular disorder	23 (4.6%)
**Unknown**	51 (9.6%)

ADPKD, autosomal-dominant polycystic kidney disease; ARPKD, autosomal-recessive polycystic kidney disease; HSP, Henoch-Scholein purpura; HUS, hemolytic-uremic syndrome.

**Table 4:** Adjusted all-cause mortality rates and hazard ratios (HRs) for mortality, stratified by primary renal disease (PRD) among end-stage renal disease (ESRD) patients, 2000–2014.

	No. of Deaths	Patient-Years (py)	Death Rate per 1000 py	Unadjusted HR (95% CI)	Adjusted HR * (95% CI)
**Glomerular disease**					
Primary GN	7	454.5	15.40	Reference	Reference
Nephrotic syndrome	5	147.33	33.94	2.10 (0.67–6.62)	1.65 (0.51–5.34)
CGN	7	589.92	11.87	0.78 (0.27–2.23)	0.81 (0.28–2.32)
Secondary GN	21	530.92	39.55	2.53 (1.03–5.95)	2.50 (1.03–6.08)
Other glomerular disease	2	79.25	25.24	1.58 (0.32–7.50)	1.37 (0.28–6.68)
**Nonglomerular disease**					
Renal hypodysplasia	10	266.17	37.57	2.93 (0.87–6.03)	1.32 (0.49–3.54)
Cystic kidney disease	1	56.75	17.62	0.98 (0.12–7.98)	0.39 (0.05–3.30)
Reflux nephropathy	2	182.0	10.99	0.65 (0.13–3.15)	0.47 (0.10–2.32)
Urologic disorder	8	106.25	75.29	4.58 (1.66–12.65)	4.77 (1.69–13.46)
Ischemic	8	192.5	41.56	2.56 (0.93–7.07)	1.64 (0.58–4.46)
Metabolic	6	46.92	127.89	6.85 (2.92–20.47)	5.57 (1.84–16.85)
Obstructive uropathy	2	68.33	29.27	1.77 (0.37–8.54)	1.50 (0.31–7.30)
Other nonglomerular disease	18	339.0	53.10	3.17 (1.32–7.59)	2.13 (0.87–5.21)
**Unknown**	10	338.08	29.58	1.89 (0.72–4.97)	1.80 (0.67–4.84)

CGN, chronic glomerulonephritis; CI, confidence interval; ESRD, end-stage renal disease; GN, glomerulonephritis; HR, hazard ratio. * Estimated from a multivariate Cox proportional hazards model with adjustment for age group, sex, initial dialysis modality, calendar year of dialysis initiation, and number of comorbidities; py, person-year; No., number.

## Supplementary Materials

The following are available online at http://www.mdpi.com/2077-0383/7/11/414/s1, Table S1: ICD-9-CM codes for primary renal diseases in children and young adults on chronic dialysis, Table S2: ICD-9-CM codes for comorbidities of children and young adults during dialysis.
