# Supplementary material for: Mortality Risks among Various Primary Renal Diseases in Children and Adolescents on Chronic Dialysis
Source: J Clin Med. 2018 Nov 5;7(11):414. doi: 10.3390/jcm7110414 (PMC6262556; doi:10.3390/jcm7110414)
Supplement: Supplementary file 1 [file jcm-07-00414-s001.pdf]

Mortality Risks among Various Primary Renal Diseases in Children and Adolescents on Chronic Dialysis

Hsin-Hsu Chou MD[1,2], Yuan-Yow Chiou MD, PhD[3], Yee-Hsuan Chiou MD[4,5], You-Lin Tain MD, PhD[6], Hsin-Hui Wang MD, PhD[7], Mei-Ching Yu MD, PhD[8], Chih-Cheng Hsu MD, PhD; [9-11] and Ching-Yuang Lin MD, PhD[12,*]

Table S1. ICD-9-CM codes for primary renal diseases in children and young adults on chronic dialysis.

Glomerular	
Acute glomerulonephritis	580.x, 581.x, 583.0-4
Chronic glomerulonephritis	582.x
Vasculitis	710.0, 287.0, 446.4
Others	583.6-9, 250.4, 238.11, 446.6
Non-glomerular	
Renal hypodysplasia	753.0, 587, 589.x
Reflux nephropathy	593.7
Urologic disorder	344.61, 596.0, 596.4-5, 599.6, 753.2x, 753.5-6
Cystic kidney disease	753.1x
Ischemic	343.x, 572.4, 584.5-7, 758.52, 758.59, 768.x, 770.88, 958.4, 995.0, 995.4, 997.01, 998.0
Metabolic	255.13, 270.0, 271.8, 272.7, 274.1, 275.49, 588.89
Others	188, 189.0, 403.x, 584.8-9, 599.8, 753.3-4, 753.7-9, 758.x, 759.5, 759.7-9

Table S2. ICD-9-CM codes for comorbidities of children and young adults during dialysis.

Comorbidity	ICD-9-CM codes
**Diabetes**	250, 357.2, 362.0x, 366.41, A18.1
**Hypertension**	401-405
**Ischemic heart disease**	410-414
**Congestive heart failure**	428.x
**Chronic liver diseases and cirrhosis**	571.x
**Stroke**	430-438
**Malignancy**	140-208
**Growth retardation**	783.2x, 783.4x
